# Cholera Deaths During Outbreaks in Uvira, Eastern Democratic Republic of the Congo, 10–35 Months After Mass Vaccination

**DOI:** 10.1093/ofid/ofae058

**Published:** 2024-02-01

**Authors:** Patrick Musole Bugeme, Hanmeng Xu, Chloe Hutchins, Juan Dent, Jaime Mufitini Saidi, Baron Bashige Rumedeka, Moïse Itongwa, Joël Faraja Zigashane Mashauri, Faraja Masembe Lulela, Justin Bengehya, Jean-Claude Kulondwa, Amanda K Debes, Iza Ciglenecki, Esperance Tshiwedi, Faida Kitoga, Tavia Bodisa-Matamu, Taty Nadège, Hugo Kavunga-Membo, Octavie Lunguya, Placide Okitayemba Welo, Jackie Knee, Daniel Mukadi-Bamuleka, Andrew S Azman, Espoir Bwenge Malembaka

**Affiliations:** Department of Epidemiology, Johns Hopkins University, Baltimore, Maryland, USA; Centre for Tropical Diseases and Global Health (CTDGH), Université Catholique de Bukavu, Bukavu, Democratic Republic of the Congo; Department of Epidemiology, Johns Hopkins University, Baltimore, Maryland, USA; Department of Disease Control, London School of Hygiene & Tropical Medicine, London, UK; Department of Epidemiology, Johns Hopkins University, Baltimore, Maryland, USA; Zone de Santé d’Uvira, Ministère de la Santé Publique, Hygiène et Prévention, Uvira, Democratic Republic of the Congo; Department of Epidemiology, Johns Hopkins University, Baltimore, Maryland, USA; Zone de Santé d’Uvira, Ministère de la Santé Publique, Hygiène et Prévention, Uvira, Democratic Republic of the Congo; Oxfam GB, Uvira, Democratic Republic of the Congo; Oxfam GB, Uvira, Democratic Republic of the Congo; Oxfam GB, Uvira, Democratic Republic of the Congo; Division Provinciale de la Santé Publique du Sud-Kivu, Ministère de la Santé Publique, Hygiène et Prévention, Bukavu, Democratic Republic of the Congo; Division Provinciale de la Santé Publique du Sud-Kivu, Ministère de la Santé Publique, Hygiène et Prévention, Bukavu, Democratic Republic of the Congo; Department of International Health, Johns Hopkins University, Baltimore, Maryland, USA; Medical Department, Médecins Sans Frontières, Geneva, Switzerland; Rodolphe Merieux INRB-Goma Laboratory, Institut National de Recherche Biomédicale (INRB), Goma, Democratic Republic of the Congo; Rodolphe Merieux INRB-Goma Laboratory, Institut National de Recherche Biomédicale (INRB), Goma, Democratic Republic of the Congo; Rodolphe Merieux INRB-Goma Laboratory, Institut National de Recherche Biomédicale (INRB), Goma, Democratic Republic of the Congo; Programme National d’Elimination de Choléra et de lutte contre les autres Maladies Diarrhéiques (PNECHOL-MD), Ministère de la Santé Publique, Hygiène et Prévention, Kinshasa, Democratic Republic of the Congo; Rodolphe Merieux INRB-Goma Laboratory, Institut National de Recherche Biomédicale (INRB), Goma, Democratic Republic of the Congo; Institut National de Recherche Biomédicale, INRB, Kinshasa, Democratic Republic of the Congo; Institut National de Recherche Biomédicale, INRB, Kinshasa, Democratic Republic of the Congo; Programme National d’Elimination de Choléra et de lutte contre les autres Maladies Diarrhéiques (PNECHOL-MD), Ministère de la Santé Publique, Hygiène et Prévention, Kinshasa, Democratic Republic of the Congo; Department of Disease Control, London School of Hygiene & Tropical Medicine, London, UK; Rodolphe Merieux INRB-Goma Laboratory, Institut National de Recherche Biomédicale (INRB), Goma, Democratic Republic of the Congo; Institut National de Recherche Biomédicale, INRB, Kinshasa, Democratic Republic of the Congo; Department of Epidemiology, Johns Hopkins University, Baltimore, Maryland, USA; Geneva Centre for Emerging Viral Diseases, Geneva University Hospitals (HUG), Geneva, Switzerland; Division of Tropical and Humanitarian Medicine, Geneva University Hospitals (HUG), Geneva, Switzerland; Department of Epidemiology, Johns Hopkins University, Baltimore, Maryland, USA; Centre for Tropical Diseases and Global Health (CTDGH), Université Catholique de Bukavu, Bukavu, Democratic Republic of the Congo

## Abstract

Our understanding of the burden and drivers of cholera mortality is hampered by limited surveillance and confirmation capacity. Leveraging enhanced clinical and laboratory surveillance in the cholera-endemic community of Uvira, eastern Democratic Republic of Congo, we describe cholera deaths across 3 epidemics between September 2021 and September 2023 following mass vaccination.

Cholera is an acute diarrheal disease that can rapidly cause severe dehydration and death without prompt and aggressive rehydration [1]. Estimates of the true burden of cholera are highly uncertain because surveillance systems often lack routine identification and testing of suspected cases and documentation of community cases and deaths is limited. In resource-constrained settings, passive, facility-based surveillance data on mortality are likely to underestimate true mortality; contributing factors include limited patient access to health facilities and incentives for underreporting [2–4]. Additionally, limited access to confirmatory laboratories for cases and deaths may lead to distorted estimates of the true cholera mortality, both at local and global levels [5]. In the city of Uvira in South Kivu (eastern Democratic Republic of the Congo [DRC]), we implemented an enhanced cholera surveillance system as part of an impact evaluation of preventive mass oral cholera vaccination campaigns conducted in 2020. The killed oral cholera vaccine (kOCV) Euvichol-plus was administered to individuals aged ≥12 months living in Uvira in 2 mass campaigns conducted in July through August and October 2020. In this study, we aimed to explore the difference in sociodemographic characteristics between cholera deaths and survivors, to estimate health facility cholera case fatality ratio (CFR), and to estimate the effectiveness of killed oral cholera vaccine against death in Uvira across 3 cholera epidemics in the first 3 years after vaccination.

## METHODS

Between 1 September 2021 and 30 September 2023 (10–35 months after the second round of vaccination), suspected cholera cases were recruited at the 2 cholera treatment facilities in Uvira: the Cholera Treatment Centre at the Uvira General Referral Hospital and Cholera Treatment Unit at the Kalundu CEPAC health center (both designated as CTC here). A suspected cholera case was any person aged ≥12 months, reporting ≥ 3 acute, watery, and nonbloody diarrheal stools within the 24 hours before hospitalization. Data on sociodemographic characteristics, clinical manifestations, including the level of dehydration based on the Global Task Force on Cholera Control guidance [6], vaccination status, and clinical outcome were collected using a structured electronic questionnaire. Written consent was obtained from participants. Rectal swabs and stool samples were collected from consenting patients. Rectal swabs were enriched for 6 to 18 hours in alkaline peptone water at ambient temperature (∼30 °C). Crystal VC Rapid Diagnostic Tests (RDTs; Arkay, India) were used to test raw stool samples and rectal swab enrichments. Samples collected after September 2022 were cultured at the CTC laboratory, whereas those collected before were tested at Rodolphe Mérieux INRB-Goma laboratory in North Kivu. In addition, stool samples from the 2021–2022 outbreak were spotted onto Whatman 903 Protein Saver Cards (Cytiva, UK) and tested by polymerase chain reaction (PCR) at Johns Hopkins University [7].

Community deaths were not systematically captured by the surveillance system, so the study team learned of suspected community cholera deaths through informal means, with no formal protocol, as described in Supplementary Table 1. Visits were organized to collect additional information on the circumstances of death, sociodemographics, clinical manifestations, and vaccination status for each suspected community cholera death. Cholera was the suspected cause of death if the deceased was aged ≥12 months and the family members reported that they experienced acute watery diarrhea in the 24 hours preceding the death, with no other cause of death reported. Biological samples were collected on arrival for deaths that occurred during the transit to the CTC.

The characteristics of study participants were compared using the Wilcoxon rank-sum and Pearson chi-squared (or Fisher exact) tests. We estimated the effectiveness of at least 1 dose of kOCV against severe cholera (defined by a culture/PCR-confirmed cholera case with severe or treatment plan C dehydration) and suspected cholera death (ie, any suspected cholera case dying within the CTC with no other cause of death identified) using the screening method, which relies on contrasts of the proportion of cases vaccinated and the vaccine coverage in the population [8], using the formula:


$$VE=1-\;\frac{PCV}{1-PCV}\;\times \;\frac{1-PPV}{PPV}$$

where VE is the vaccine effectiveness, PCV the proportion of vaccinated cholera cases, and PPV the population vaccination coverage. We estimated confidence intervals for VE based on a logistic regression model, as previously proposed [8].

As vaccine coverage declined over time because of population movement, we estimated a weighted vaccination coverage, based on a log-linear regression of data from 3 representative vaccine coverage surveys conducted 11, 19, and 29 months after vaccination [9].

## RESULTS

**Figure 1.**
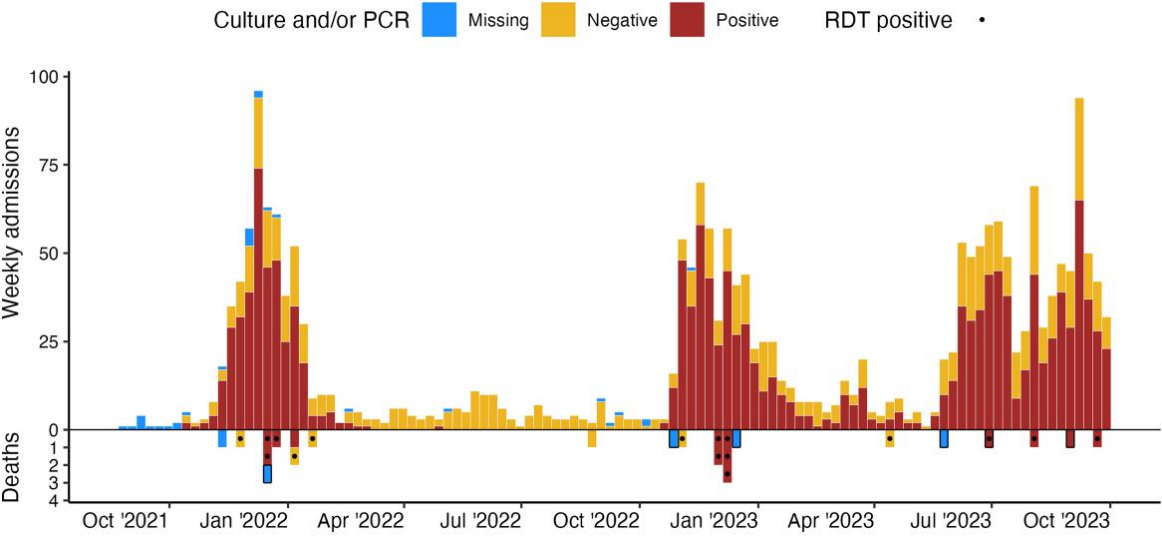
Weekly cholera incidence (top) and death (bottom) by culture or polymerase chain reaction confirmation results in Uvira, 1 September 2021 to 30 September 2023. Before 10 September 2022, culture was performed with a significant lag at an external reference laboratory (INRB Goma) from wet filter papers (stool or rectal swab enrichments suspended in saline), likely leading to reduced sensitivity for detection of *Vibrio cholerae* O1. From 10 September 2022, culture has been performed onsite in Uvira. Rapid diagnostic tests (a mix of O1 and O1/O139 tests) were also used throughout the study. For this reason, we show enriched rapid test–positive results as dots to help understand which deaths have more laboratory data supporting *V cholerae* O1 as the causative agent. Community deaths are highlighted with black boxes.

During the 25-month study period, 2209 suspected cholera cases were admitted to the CTCs, of which 1312 (61.9%) were RDT-positive and 1460 (67.3%) cases were confirmed by culture or PCR (Table 1, Figure 1). In the same period, 24 suspected cholera deaths were reported, of which 18 (75%) occurred within CTCs, with a facility-based suspected CFR of 0.8% (18/2209).

**Table 1.** Characteristics of Patients With Suspected Cholera who Died and Survived in the Cholera Treatment Facilities, Uvira, September 2021 to September 2023

Characteristic	Overall, N = 2209	Suspected Cholera Deaths, N = 18	Suspected Cholera Cases (Survivors), N = 2191	*P* Value	Suspected Case Fatality Ratio
Age (y), median (IQR)	17.0 (7.0, 34.0)	40.0 (21.0, 67.8)	17.0 (7.0, 34.0)	<.001	0.81%
Age group (y)				<.001	
<5	384 (17.4%)	1 (5.6%)	383 (17.5%)		0.26%
5–14	608 (27.5%)	1 (5.6%)	607 (27.7%)		0.16%
15–59	1024 (46.4%)	8 (44.4%)	1016 (46.4%)		0.78%
≥60 y	193 (8.7%)	8 (44.4%)	185 (8.4%)		4.12%
Sex				.873	
Female	1063 (48.1%)	9 (50.0%)	1054 (48.1%)		0.85%
Male	1146 (51.9%)	9 (50.0%)	1137 (51.9%)		0.79%
Health facility				.758	
CTC (Uvira Referral Hospital)	1806 (81.8%)	16 (88.9%)	1790 (81.7%)		0.89
CTU (Kalundu CEPAC health center)	403 (18.2%)	2 (11.1%)	401 (18.3%)		0.50%
Level of dehydration on admission^a^				.002	
Mild to moderate	1060 (48.0%)	2 (11.1%)	1058 (48.3%)		0.19%
Severe	1149 (52.0%)	16 (88.9%)	1133 (51.7%)		1.39%
Time from symptoms onset to hospitalization, d				.253	
<1	1209 (54.9%)	9 (50.0%)	1200 (54.9%)		0.74%
1	776 (35.2%)	9 (50.0%)	767 (35.1%)		1.16%
≥2	219 (9.9%)	0 (0.0%)	219 (10.0%)		0%
Missing	5	0	5		
Duration of hospitalization, d				<.001	
<1	130 (6.0%)	7 (38.9%)	123 (5.7%)		5.38%
1	542 (24.8%)	6 (33.3%)	536 (24.7%)		1.11%
≥2	1512 (69.2%)	5 (27.8%)	1507 (69.6%)		0.33%
Missing	25	0	25		
Comorbidities^b^				.490	
No	1449 (91.9%)	7 (87.5%)	1442 (92.0%)		0.48%
Yes	127 (8.1%)	1 (12.5%)	126 (8.0%)		0.79%
Missing	633	10	623		
Sought care at another health facility				.746	
No	1875 (84.9%)	15 (83.3%)	1860 (84.9%)		0.80%
Yes	334 (15.1%)	3 (16.7%)	331 (15.1%)		0.90%
Treated at home or pharmacy				.557	
No	1075 (48.7%)	10 (55.6%)	1065 (48.6%)		0.93%
Yes	1134 (51.3%)	8 (44.4%)	1126 (51.4%)		0.71%
Use of antibiotics before admission^d^				.232	
No	1163 (52.6%)	12 (66.7%)	1151 (52.5%)		1.03%
Yes	1046 (47.4%)	6 (33.3%)	1040 (47.5%)		0.57%
APW-enriched RDT				.081	
Negative	807 (38.1%)	3 (17.6%)	804 (38.2%)		0.37%
Positive	1312 (61.9%)	14 (82.4%)	1298 (61.8%)		1.07%
Missing	90	1	89		
Culture/PCR-confirmed cholera				.818	
Negative	751 (34.5%)	6 (35.3%)	703 (34.5%)		0.80%
Positive	1428 (65.5%)	11 (64.7%)	1417 (65.5%)		0.77%
Missing	30	1	29		
Vaccination status				>.999^c^	
Not vaccinated	1688 (81.0%)	11 (84.6%)	1677 (80.9%)		0.65%
At least 1 dose	397 (19.0%)	2 (15.4%)	395 (19.1%)		0.50%
Missing	124	5	129		

Data are n (proportion) unless otherwise specified.

Abbreviations: APW, alkaline peptone water; CTC, cholera treatment center; CTU, Cholera Treatment Unit; IQR, interquartile range; PCR, polymerase chain reaction; RDT, Rapid Diagnostic Test.

^a^The level of dehydration assessed using the Global Task Force on Cholera Control (GTFCC) guidance [6].

^b^Comorbidities refer to self-reported diabetes, hypertension, or HIV infection (Supplementary Table 1). *P* values are derived from Pearson chi-squared test or Fisher exact test comparing attributes between survivors and those who died, denoted by ^c^. A confirmed cholera case was defined by a positive PCR or culture result. A negative case was confirmed by negative PCR and culture, or by a negative culture only result when PCR was not done.

^d^Use of antibiotics was defined as consumption, after medical prescription or self-medication, of at least 1 of the following antibiotics: amoxycillin, azithromycin, cefixime, ceftriaxone, cefuroxime, chloramphenicol, ciprofloxacin, cotrimoxazole, doxycycline, flucloxacillin, levofloxacin, metronidazole, penicillin, tetracycline, tinidazole, and nalidixic acid.

Fourteen (82.4%) of the 17 health facility deaths in which a sample was collected tested positive for cholera by RDT and 11 (64.7%) by culture or PCR. The overall culture/PCR-confirmed facility-based CFR was 0.77% (11/1428), though this was significantly higher in participants aged ≥ 60 years (5.4% [6/105], *P* < .001) compared with younger participants. The suspected cholera CFR in this age group (≥60 years) was 5.5% (11/191) (Supplementary Tables 2 and 3).

Those dying with suspected cholera tended to be older than suspected cases who survived, with a median age of 40.0 (interquartile range 21.0–67.8 years), more than double that of survivors (median [interquartile range]: 17.0 [7.0–34.0] years; *P* < .001), with no difference by sex (Table 1). Suspected cases who died were more likely to have been admitted with severe dehydration compared with survivors (88.9% vs 51.7%, *P* = .002). A comparison of characteristics of confirmed cholera cases and deaths (Supplementary Table 2) yielded qualitatively similar results to those based on clinical case definitions (Table 1). Eleven (61.1%) health facility deaths occurred after at least 1 day of hospitalization. Two of the facility deaths with known vaccination status (n = 13), both culture-positive, were reported to have received 1 dose of oral cholera vaccine during the 2020 vaccination campaign, but we were unable to confirm these with vaccination cards.

Overall, 19% (n = 397) of patients admitted to CTCs reported having received at least of dose of kOCV (Supplementary Table 4). We estimated a vaccine effectiveness for at least 1 dose of kOCV of 78.4% (95% confidence interval, 74.2–82.4) against severe culture/PCR-confirmed cholera (dehydration plan C) and 85.5% (95% confidence interval, 49.0–97.7) against death from all-cause diarrhea (suspected cholera death). The method we used to estimate vaccine effectiveness relies heavily on assumptions about the vaccine coverage in the community, and even when assuming a 10-percentage point lower vaccination coverage in the population than measured from cross-sectional surveys, the estimated VE against suspected cholera death remained substantial (>70%) (Supplementary Figure 1).

The age of community deaths ranged between 4 and 74 years (median: 38 years), with half (n = 3) being female. Two community deaths had a rectal swab collected and tested positive for cholera by RDT and culture.

## DISCUSSION

The 0.8% facility-based CFR for suspected cholera cases observed from this passive surveillance system aligns with the World Health Organization target of <1% and was lower than that reported between 2008 and 2017 in DRC hotspot health zones (1.1%) [10]. Although we captured some community deaths, the true cholera mortality burden in Uvira is likely higher than reported here because of lack of robust community-based surveillance and the number of private health facilities that are not integrated into the official surveillance system. The limited existing studies comparing the reported number of facility deaths with those occurring in both facilities and the community suggest this gap is large [2–4, 11]. A study from rural Kenya showed that although suspected cholera cases were underreported by 37%, suspected cholera deaths were underreported by 200%, implying a 52% underestimate of the community CFR [2–4, 11]. In Cameroon, 44% of suspected cholera deaths occurred in the community or during transit to a treatment unit [4]. During the 2010–2011 cholera outbreak in Haiti, community surveys led to almost a 3-fold higher death toll in certain areas compared with official facility-based estimates [3].

A previous report from across DRC suggested that 20.8% of the suspected cholera deaths during the 2008–2017 period were among those aged <5 years, a stark contrast to the 6.6% in our study [10]. This difference may be a result of several factors including the systematic application of case definitions in Uvira (ie, improved specificity) and differences in health-seeking behaviors and/or in the quality of care given to young patients in Uvira.

Almost half of the health facility deaths (8/18) occurred among patients aged at least 60 years, leading to an unacceptably high age group–specific CFR (4.0%). Moreover, 61.1% of deaths happened after ≥ 1 day of hospitalization. This may be because clinical assessment and management of severe dehydration is challenging in the elderly, who might have comorbidities requiring a slower rehydration pace to avoid fluid overload (Supplementary Table 2). Complications related to underlying comorbidities such as cardiovascular diseases, diabetes mellitus, anemia, or malnutrition may be overlooked in cholera treatment centers, particularly in the middle of an outbreak because rehydration is the priority. CTCs in most humanitarian settings, including in our study site, are not sufficiently equipped for an adequate assessment and management of other health conditions cholera patients may present with, including chronic morbidities. Ensuring that CTCs offer comprehensive and person-centered care (as opposed to solely dehydration-centered care) might contribute to the reduction of cholera CFR, particularly among the elderly.

The CFR among patients who reported use of antibiotics before admission to CTCs was almost half that of patients who did not use antibiotics, although the difference was not statistically significant (Table 1). This finding is not surprising because antibiotics are known to reduce the duration and severity of cholera [12]. Larger scale studies are needed to better understand whether and how antibiotics use can contribute to reducing mortality risk.

Our findings indicate that the only kOCV available in the global stockpile at the time of writing this manuscript (Euvichol-plus) is highly protective against severe cholera and death. The few available studies reporting kOCV effectiveness against severe cholera point to similar levels of protection, ranging from 73% (23–91%) 4 to 24 months after vaccination in Haiti [13] to 48% (16–67%) in the fourth year after vaccination in Bangladesh [14]. Despite the relatively small number of deaths limiting more stratified analyses, this study provides the first insights into kOCV effectiveness against death and suggests that large-scale deployment of kOCVs in preventive vaccination campaigns may substantially contribute to achieving the Global Task Force on Cholera Control's goal of reducing cholera mortality by 90% by 2030 [15]. We used this observational study to estimate the VE against cholera death using the screening method, which relies on estimates of vaccine coverage in cases (ie, cholera deaths) and the population from which these cases came from. Although we used weighted estimates from 3 population-representative coverage surveys, it is possible that the true source population for cholera cases/deaths coming into the treatment centers has a different overall coverage to the general population of the city. Sensitivity analyses suggested our estimates were robust to modest differences in coverage compared with our main assumption, although we cannot exclude the possibility of bias. Our estimates of VE represent the protection conferred against both getting cholera and dying and based on the small effect size of the association between vaccine receipt and survival among cases within the CTC, we believe this effect is largely driven by protection against severe disease.

## CONCLUSION

Although the public health community strives to improve the cholera surveillance [16], describing the magnitude and drivers of community and confirmed facility deaths is critical to help correctly target those most at risk and improve patient care and estimates of cholera burden. This study provides a unique insight into confirmed cholera mortality and vaccine protection in a resource-constrained and endemic setting. Studies with larger sample sizes, including community surveillance of deaths and in settings with different levels of endemicity, are needed to confirm and expand on our findings.

## Supplementary Material

ofae058_Supplementary_Data

## Supplementary Data

Supplementary materials are available at *Open Forum Infectious Diseases* online. Consisting of data provided by the authors to benefit the reader, the posted materials are not copyedited and are the sole responsibility of the authors, so questions or comments should be addressed to the corresponding author.
